# Supplementary material for: Digital Health Literacy of People with Intellectual Disabilities: A Scoping Review to Map the Evidence
Source: Int J Environ Res Public Health. 2025 Nov 19;22(11):1748. doi: 10.3390/ijerph22111748 (PMC12651974; doi:10.3390/ijerph22111748)
Supplement: Supplementary file 1 [file ijerph-22-01748-s001.zip › Supplemental File S2 Search strategies.pdf]

Supplemental File S2

Overview of the search strategies

Search conducted on 04th January 2024

Search Strategy:

#	database	Search string	Results
1	PubMed	(Intellectual* disab* OR learning disab* OR cognitive* impair* OR developmental disab* OR intellectual handicap* OR mental* retard* OR mental* abnormal* OR down syndrome) AND (digital health literacy OR ehealth literacy OR electronic health literacy OR media health literacy OR mobile health literacy OR digital competenc* OR media skill* OR media competenc* OR digital literacy OR digital divide OR digital gap* OR digital inequalit* OR digital inclusion OR eHealth Services OR internet use OR social media)	2480
2	Cinahl	(Intellectual* disab* OR learning disab* OR cognitive* impair* OR developmental disab* OR intellectual handicap* OR mental* retard* OR mental* abnormal* OR down syndrome) AND (digital health literacy OR ehealth literacy OR electronic health literacy OR media health literacy OR mobile health literacy OR digital competenc* OR media skill* OR media competenc* OR digital literacy OR digital divide OR digital gap* OR digital inequalit* OR digital inclusion OR eHealth Services OR internet use OR social media)	357
3	Eric	Intellectual* disab* OR learning disab* OR cognitive* impair* OR developmental disab* OR intellectual handicap* OR mental* retard* OR mental* abnormal* OR down syndrome anywhere and "digital health literacy OR ehealth literacy OR electronic health literacy OR media health literacy OR mobile health literacy OR digital competenc* OR media skill* OR media competenc* OR digital literacy OR digital divide OR digital gap* OR digital inequalit* OR digital inclusion OR eHealth Services OR internet use OR social media"	1144
4	Web of science	(Intellectual* disab* OR learning disab* OR cognitive* impair* OR developmental disab* OR intellectual handicap* OR mental* retard* OR mental* abnormal* OR down syndrome) AND digital health literacy OR ehealth literacy OR electronic health literacy OR media health literacy OR mobile health literacy OR digital competenc* OR media skill* OR media competenc* OR digital literacy OR	4696

		digital divide OR digital gap* OR digital inequalit* OR digital inclusion OR eHealth Services OR internet use OR social media	
5	Livivo	((Intellectual* disab* OR learning disab* OR cognitive* impair* OR developmental disab* OR intellectual handicap* OR mental* retard* OR mental* abnormal* OR down syndrome) AND TI=(digital health literacy OR ehealth literacy OR electronic health literacy OR media health literacy OR mobile health literacy OR digital competenc* OR media skill* OR media competenc* OR digital literacy OR digital divide OR digital gap* OR digital inequalit* OR digital inclusion OR eHealth Services OR internet use OR social media)) AND PY=2009:2023	230
6	Wiley	"Intellectual* disab* OR learning disab* OR cognitive* impair* OR developmental disab* OR intellectual handicap* OR mental* retard* OR mental* abnormal* OR down syndrome" anywhere and "digital health literacy OR ehealth literacy OR electronic health literacy OR media health literacy OR mobile health literacy OR digital competenc* OR media skill* OR media competenc* OR digital literacy OR digital divide OR digital gap* OR digital inequalit* OR digital inclusion OR eHealth Services OR internet use OR social media"	468
7	Psyndex	(geistige Behind* OR Intellektuelle Behind* OR intellektuelle beeinträcht* OR menschen mit lernschwierigkeit*) AND (digital health literacy OR digitale Gesundheitskomptenz OR digitale Kompetenz* OR Medienkomp* OR digital divide OR digitale Kluft OR digitale ungerechtigkeit* OR digitale inklusion OR internetnutzung OR social media OR digitale Angebote)	6
8	Rehadat	(geistige Behind* OR Intellektuelle Behind* OR intellektuelle beeinträcht* OR menschen mit lernschwierigkeit*) AND (digital health literacy OR digitale Gesundheitskomptenz OR digitale Kompetenz* OR Medienkomp* OR digital divide OR digitale Kluft OR digitale ungerechtigkeit* OR digitale inklusion OR internetnutzung OR social media OR digitale Angebote) bzw. (geistige Behind* ODER Intellektuelle Behind* ODER intellektuelle beeinträcht* ODER menschen mit lernschwierigkeit*) UND (digital health literacy ODER digitale Gesundheitskomptenz ODER digitale Kompetenz* ODER Medienkomp* ODER digital divide ODER digitale Kluft ODER digitale ungerechtigkeit* ODER digitale inklusion ODER internetnutzung ODER social media ODER digitale Angebote)	3
9	Fachportal Pädaogig	((((((((((((Freitext: GEISTIGE und BEHINDERUNG) oder (Freitext: INTELLEKTUELLE und BEHINDERUNG)) oder (Freitext: INTELLEKTUELLE und BEEINTRAECHTIGUNG)) oder (Freitext: LERNSCHWIERIGKEIT)) und (Freitext: DIGITALE und GESUNDHEITKOMPETEZ)) oder (Freitext: DIGITAL und HEALTH und LITERACY)) oder (Freitext: DIGITALE und KOMPETENZ)) oder (Freitext:	2815

		MEDIENKOMPTENZ)) oder (Freitext: DIGITAL und DIVIDE)) oder (Freitext: DIGITALE und KLUFT)) oder (Freitext: DIGITALE und UNGERECHKTIGKEIT)) oder (Freitext: DIGITALE und INKLUSION)) und (Sprache: deutsch oder englisch oder andere)) und (Datenquelle: "FIS Bildung" oder "Library of Congress" oder "Casalini libri" oder ERIC oder "EBSCOhost ebooks" oder "BBF 1945-1993" oder "Online Contents" oder FID-Nationallizenzen oder BASE)	

Search conducted on 12th February 2024 (as access authorization to the databases had to be obtained first)

Search Strategy:

#	database	Search string	Results
10	Psycindex	Intellectual* disab* OR learning disab* OR cognitive* impair* OR developmental disab* OR intellectual handicap* OR mental* retard* OR mental* abnormal* OR down syndrome anywhere and "digital health literacy OR ehealth literacy OR electronic health literacy OR media health literacy OR mobile health literacy OR digital competenc* OR media skill* OR media competenc* OR digital literacy OR digital divide OR digital gap* OR digital inequalit* OR digital inclusion OR eHealth Services OR internet use OR social media"	405
11	Carelit	(geistige Behind* OR Intellektuelle Behind* OR intellektuelle beeinträcht* OR menschen mit lernschwierigkeit*) AND (digital health literacy OR digitale Gesundheitskomptenz OR digitale Kompetenz* OR Medienkomp* OR digital divide OR digitale Kluft OR digitale ungerechtigkeit* OR digitale inklusion OR internetnutzung OR social media OR digitale Angebote)	24

Search conducted on 27th April 2024 for grey literature

Search Strategy:

#	database	Search string	Results
	Google scholar	Intellectual* disab* OR learning disability* OR cognitive* impaired* OR developmental disability* OR intellectual handicap* OR mental* retard* OR mental* abnormal* OR down syndrome AND digital health literacy OR ehealth literacy OR electronic health literacy OR media health literacy OR mobile health literacy OR digital competence* OR media skill* OR media competence* OR digital literacy	1210
