# Supplementary material for: Digital Health Literacy of People with Intellectual Disabilities: A Scoping Review to Map the Evidence
Source: Int J Environ Res Public Health. 2025 Nov 19;22(11):1748. doi: 10.3390/ijerph22111748 (PMC12651974; doi:10.3390/ijerph22111748)
Supplement: Supplementary file 1 [file ijerph-22-01748-s001.zip › Supplemental File S3 Overview included studies.pdf]

Supplemental File S3

Overview of the included studies

Author	year	Country	Challenges addressed and objective	Study design	Sample	Setting	Theoretical foundation	Digital health literacy relevance
Hall et al.	2011	UK	• people with ID have poor access to health care<br>• virtual reality has been shown to support learning in people with intellectual disabilities in a variety of ways and to provide a safe setting in which they can practice activities that might not be possible in the real world<br>• study assessed the acceptability, usability, and potential utility of a virtual reality experience as a means of providing health care-related information	intervention and qualitative research design	20 people aged between 20 and 80 years with mild to severe intellectual disabilities	community setting	not mentioned	• improve health care access in real-life via digital learning<br>• providing easily accessible digital health information using a multimodal learning approach<br>• enhancing understanding and memory retention of health information
Salmeron et al.	2016	Spain	• concerns raised regarding the potential limitations of people with ID to deal with untrustworthy information sources on the internet<br>• in an experiment it was assessed how adult students with ID evaluated recommendations in Internet forums	quantitative research	40 students with intellectual disabilities (average age 19 years ranging from 17 to 23 years old)	students from a vocational training center for people with special needs from a mid-size city, and a control group of 59 fifth-grade students from a regular school	not mentioned	• evaluating the trustworthiness of information sources from internet forums regarding health<br>• comparing different groups varying cognitive abilities of critical thinking in assessing online health-related content.
Arachchi et al.	2017	Australia and Sri Lanka	• unclear, how to design an engaging eLearning environment that integrates the usability with learning<br>• aims to explore the applicability of learning theories along with usability guidelines in designing an eLearning environment for people with ID<br>• Discuss theories to develop guidelines for an eLearning-platform to access online health information<br>• case study: eLearning module describes and teaches how to access health information	theoretical approach	not suitable	not suitable	Behavioral Learning Theory, Social Cognitive Theory, Constructivist Learning Theory, Interactions in eLearning	• eLearning designs that match users' cognitive abilities<br>• in this way improving access to health information and resources through eLearning platform<br>• teaching and learning theories enhances the educational framework for the effective target-group orientated communication of digital health information
Chadwick et al.	2017	UK	• people with intellectual disabilities are not accessing the Internet to the same degree as people without intellectual disabilities<br>• Issues of safety, risk and protection online have yet to be adequately investigated, and these currently serve as reasons given for hindering people from gaining	quantitative research	66 members of the general population	not suitable	not mentioned	• mention unequitable access to online information<br>• results underscore the importance of understanding how these views of supporters can impact online engagement for individuals with

			online access<br>• aimed to gauge the views people without intellectual disabilities have of risks and benefits of using the Internet for themselves and for people with intellectual disabilities and to compare self-ratings of risk and benefits to ratings for people with intellectual disabilities					intellectual disabilities<br>• digital health information is mentioned as a positive connotation
Sheehan & Hassiotis	2017	UK	• people with ID experience high rates of mental illness<br>• they mostly excluded from the development and implementations of new interventions<br>• they could benefit from the development of novel therapies<br>• aim to describe a need orientated digital inclusion strategy	literature review	not suitable	not suitable	not mentioned	• addressing effective digital health interventions to improve understanding of mental health and vocational opportunities<br>• characteristic challenges of people with intellectual disabilities using digital technologies for managing health issues are discussed
Watfern	2019	Australia	• e-mental health programs are underexplored<br>• aim was to examine the feasibility and acceptability of an e-mental health program via interviews and focus groups<br>• pragmatic goal of informing further development of the website, along with strategies for its dissemination and implementation	intervention and qualitative evaluation (interviews and focus groups)	36 people with intellectual disabilities and nine support workers	connected to disablility service providers	not mentioned	• potential of e-mental health programs to address barriers to accessing mental health services was addressed<br>• qualitative research enhances understanding of user experiences<br>• different support needs were identified and discussed taking in account the social environment to get access to digital health information.
Frielink et al.	2021	Netherland	• use of eHealth in support for daily functioning of service users with intellectual disabilities are a rather unexplored domain<br>• aim was to identify the a) level of familiarity, b) advantages/disadvantages, and c) facilitating/ impeding factors for the use of eHealth in support for daily functioning via four focus groups	qualitative research	8 people with mild to borderline ID (IQ 50-85), four relatives of people with intellectual disabilities and four professionals	ID service in the southern part of the Netherlands which offered residential homes, 24-hour community residences, ambulant support at clients' own homes, and day care facilities	not mentioned	• identifying the level of familiarity with eHealth applications to enable effective access to health information<br>• discussing benefits and risks also concerning health information from users' perspective
Vetter et al.	2022	Germany	• health literacy is not target-group orientated conceptualized for people with intellectual disabilities<br>• Following research question was answered: Which dimensions influence the health literacy of people with intellectual disabilities?<br>• health literacy dimensions are analysed	qualitative research	38 interviews with people with mild to moderate intellectual disabilities from two different research projects (secondary data analysis)	inpatient and outpatient integration assistance	health literacy, **digital health literacy is mentioned by name**	• outlined dimensions, including "digital interaction spaces," directly link to digital health literacy, the role of digital information is discussed

Kuruppu Arachchi et al.	2023	Australia and Sri Lanka	• little is known about how people with intellectual disabilities interact with critical online information, such as health information,<br>• aim is to explore the current and desired use of web-search, particularly for health information, by adults with intellectual disability via structured interviews	qualitative research	39 participants	in supported employment or attending day centers in Australia	health literacy	• emphasizes the importance of web searches for health information<br>• identifying participants' preference for engaging and accessible formats of online health information<br>• based on wishes to interact with health information online, the study contributes to broader discussions of digital health literacy
Dam et al.	2023	Austria	• challenges exist including difficulties in comprehending written health information, communication barriers with healthcare professionals, and limited inclusion in health education research,<br>• aim to improve knowledge transfer of health information for people with intellectual disabilities by creating a website prototype.<br>• based on a literature review unstructured interviews were conducted with two focus groups people with intellectual disabilities and their caretakers to elicit relevant topics and ensure the quality,<br>• comprehensibility, and usability of a website prototype from an accessible design perspective as well as from a learning and support standpoint are discussed	intervention based on qualitative research and qualitative evaluation	20 adults working in sheltered workshops (mild to moderate intellectual development disorder) according to ICD-11	sheltered workshops (mild to moderate intellectual development disorder) according to ICD-11	health literacy	• project aims to improve digital access to health information, usage of websites and enhance health literacy<br>• various learning styles aiming enhances understanding of online health information
Savage	2023	USA	• interventions to support healthier nutrition habits are lacking<br>• technology may increase exposure and support<br>• little is known about use of health apps, barriers challenges and concerns about universal designs and need for additional support<br>• a developed app addressing diet, health, and fitness goals were tested	intervention and qualitative evaluation	2 adults with mild intellectual disabilities (Autism & Trisomie 21) and 1 with a moderate intellectual disability (Trisomie 21)	participant's home and their local and preferred grocery store	not mentioned	• explores the use of a health app to manage health issues (nutrition habits)<br>• empowers individuals to take control of their health habits<br>• showcases how digital resources can effectively influence health behaviors via online health information
