# Supplementary material for: Digital Health Literacy of People with Intellectual Disabilities: A Scoping Review to Map the Evidence
Source: Int J Environ Res Public Health. 2025 Nov 19;22(11):1748. doi: 10.3390/ijerph22111748 (PMC12651974; doi:10.3390/ijerph22111748)
Supplement: Supplementary file 1 [file ijerph-22-01748-s001.zip › Supplemental File S4 Overview factors.pdf]

Supplemental File S4

Overview of the situational, personal, and environmental factors

		Situational	Personal		Environmental	
**Author**	**year**	**Health concerncs / Health topic**	**Socio-demographics**	**Individual skills**	**Media environment**	**Social environment**
Hall et al.	2011	healthcare information in general not a specific topic or concern	not mentioned otherwise than sample description	not mentioned otherwise than sample description or in general ("although skill levels varied")	people with intellectual disabilities may have trouble operating multifunction control devices due to problems in remembering which device achieves which task, or they may experience fine-motor difficulties, which could leave them feeling frustrated and demotivated	six volunteer psychology graduates, who had been given rudimentary instruction on the use of the virtual world acted as facilitators to the delivery of the virtual environment.
Salmeron et al.	2016	generally, misinformation in online forums	not mentioned otherwise than sample description	people with intellectual disabilities typically present a level of reading comprehension that is several years below their chronological age	readability indices indicated that texts were appropriate for fifth graders.	in the practice forum, the research assistants responded to questions regarding the procedure until students felt confident with the task
Arachchi et al.	2017	health information in general not a specific topic or concern	not mentioned otherwise than sample description	not mentioned	theoretical framework of guidelines for learner centered interaction design of eLearning environments and a functional model for eLearning design that integrate learning theories were developed	importance of training programs (basic training among low-performance groups and advanced training on essential functions) are needed, results showed that while the training program had increased the participants' interest in using computers, there remained the need for continued training on Internet use, with close support
Chadwick et al.	2017	health information in general not a specific topic or concern	not mentioned	not mentioned	not mentioned	influence of supporter's internet experience is discussed
Sheehan & Hassiotis	2017	digital mental health in general	more time to learn new skills is necessary for using technology, general understanding that younger generations	not mentioned	the text stresses the importance of "universal design" to optimize accessibility of digital technologies and discuss this for a heteregenous group like people with intellectual disabilities (principles are inconsistently)	It discusses the attitudes and behaviors of family members or professional carers, which can either enable or discourage technology use and mentions the need for

			('digital natives') are more capable, skills can be lost if digital use is not actively promoted			appropriate training and ongoing support
Watfern	2019	common mental health problems	not mentioned otherwise than sample description	not mentioned	participants provided feedback about the accessibility of information on the website, particularly considering the different types of needs that user may have when engaging with textual (easy-read English) and audio-visual information as well as log in acquirements, navigation, activities and dilemmas.	sensitive support guiding someone through the website could perhaps help alleviate some of this anxiety of not knowing, support workers expressed a tension between wanting to enable people to access the website independently as much as possible versus acknowledging and even encouraging people to engage with support as they use the site
Frielink et al.	2021	in general eHealth	eHealth can be harder for older individuals who did not grow up with technology	not mentioned	person-orientation is emphasized: "It is important to make a decision beforehand regarding which eHealth applications you want to use... So, you need to consider that on a case-by-case basis, to see what suits that particular person. And not what suits a whole group, or what suits a whole region."	person-orientation is emphasized: "It is crucial that the individual needs and possibilities of each service user are the starting point... Involving all stakeholders, for example by explaining what is going to happen... is important."
Vetter et al.	2022	in general health literacy	in general, that age, gender, language and education can have an effect on health literacy	not mentioned	not mentioned	in general, that the social context is important for health literacy
Arachchi et al.	2023	in general health information (but it was stated most popular health information interest was related to diet and additionally motivation to search for heath information by discussions with their doctors, family members or advertising)	not mentioned otherwise than sample description	not mentioned	participants wish that the designers will address their requirements in future designs of online health information e.g., voice recognition	participants expecting support mentioned: 'would love to learn computer', 'If I had help to do it', with 'Step by step guidelines'
Dam et al.	2023	specific health topics mentioned include "nutrition," "medical	significant barrier concerning the comprehension of	not mentioned	various points regarding usability, accessibility, and the importance of adapting content to the target audience are discussed.	not mentioned

		specialization," "patient information," and "first aid."	written information in any media since they are mostly provided in high complexity				
Savage	2023	Specific health topic is nutrition habits.	not mentioned otherwise than sample description	not mentioned otherwise than sample description all participants reported they were comfortable or very comfortable with technology	not mentioned		future research should investigate providing additional supports in similar situations such as a support coach or a peer group who are working towards similar goals
