# Supplementary material for: Digital Health Literacy of People with Intellectual Disabilities: A Scoping Review to Map the Evidence
Source: Int J Environ Res Public Health. 2025 Nov 19;22(11):1748. doi: 10.3390/ijerph22111748 (PMC12651974; doi:10.3390/ijerph22111748)
Supplement: Supplementary file 1 [file ijerph-22-01748-s001.zip › Supplemental File S5 Overview main results challenges adaptations.pdf]

Supplemental File S5

Overview of the main results, specific challenges and needs-orientated adaptations

author	year	main results	specific challenges	needs-oriented adaptations
Hall et al.	2011	Participants engaged immediately with the virtual environment and maintained good concentration. None had physical difficulty using the keyboard. Most improved their keyboard skills during exposure, though a few remained underdeveloped. With facilitator support, limited skills did not hinder engagement or decision-making. Participants were aware of using a computer, but this did not reduce immersion. All recalled some accurate health information, though amounts varied. Both interview parts produced some confabulated or self-added details, which increased when prompted by screenshots—some even "remembered" unvisited areas. Two months later, a focus group showed participants still retained memories	People with intellectual disabilities may struggle to understand and retain information needed for treatment decisions. Health information is usually provided through leaflets or storybooks. Existing research on virtual reality with this group mainly focuses on skill training, rehabilitation, exercise, or leisure, mostly involving younger individuals in institutional settings.	not mentioned
Salmeron et al.	2016	In this experiment, it was assessed how students with intellectual disabilities evaluate recommendations including health topics in social question and answer forums (SQA), as compared to students matched on age and verbal mental age. The main results are described in specific challenges and needs orientated challenges columns.	The results show that people with intellectual disabilities differ from control groups in how they use source information when recommending whether to follow advice in SQA. Regardless of authorship or evidence, they tend to encourage following the advice and rarely justify it by citing expert sources. Instead, they refer to external figures like teachers or parents and often add unrelated information. Control groups, however, favor expert advice and base their explanations on the forum discussion. These findings suggest not a delay, but an atypical development of source evaluation skills.	People with intellectual disabilities need targeted support to critically assess source credibility in SQA. Promoting social network use alongside specific training could help them overcome difficulties in interpreting complex text discussions.
Arachchi et al.	2017	A functional model was developed to apply learning theories in eLearning design, resulting in guidelines for learner-centered environments for people with intellectual disabilities. The tested module teaches how to access health information while improving Internet and navigation skills. Designing accessible eLearning requires attention to both technological usability and psychological suitability. This article proposes design guidelines that integrate learning principles with usability criteria to meet learners' needs.	General challenges are mentioned. For example: "The main focus of the process of designing eLearning modules for people with intellectual disabilities should be guided by minimizing the cognitive capacity required by the learner to interact with the system and the content in order to maximize the cognitive resources available for the learning process."	Designing eLearning modules for people with intellectual disabilities should focus on minimizing the cognitive effort required to use the system and content, maximizing resources for learning. The developed framework provides learner-centered interaction design guidelines that consider

				individual needs and key interactions: Learner-Interface, Content-Interface, Interface-Interface, Teacher/Tutor-Interface, and Tutor-Learning.
Chadwick et al.	2017	For people with intellectual disabilities, online social and support activities were seen as most beneficial, offering ways to engage with groups, maintain friendships, join support communities, and access advice on topics like benefits, health, and relationships. The least likely risks included antisocial behaviors such as bullying, posting harmful content, or online gambling, as well as being stalked or experiencing health issues from excessive social media use. Personal risks like grooming or falling for online scams were also rated low. Having children in the household was linked to higher perceived online benefits for this group.	The perception that this group of people may be more vulnerable in experiencing risk online could lead to less support to get online and hence to lower usage of the Internet. The role of the carer/supporter is therefore paramount when attempting to maximize the user's positive engagement with the Internet.	For people with intellectual disabilities, online social and support activities were seen as most beneficial, offering opportunities to connect with social groups, maintain friendships, join support networks, and access advice on benefits, health, and relationships.
Sheehan & Hassiotis	2017	Modern technology could transform mental healthcare, offering more effective and affordable treatments through digital interventions. However, evidence on their effectiveness and safety is limited, and people with intellectual disabilities have been largely overlooked. Although they are willing and able to use digital tools, barriers to access often lead to exclusion and reinforce existing health inequalities. Applying universal design principles can improve accessibility for people with intellectual disabilities and others with cognitive, sensory, or literacy challenges. Current research rarely includes this group, yet findings show they can engage successfully with digital technologies when given proper opportunities.	Online health information is largely unregulated and often of poor quality. While this affects all users, people with intellectual disabilities may be more vulnerable to misinformation. Universal design ('design for all') aims to create products and environments usable by everyone without adaptation, but even websites made for people with intellectual disabilities apply these principles inconsistently.<br>Common challenges include cognitive, physical, and sensory limitations; lack of training, support, and stability; frequent interface changes; economic and attitudinal barriers; self-exclusion; protective attitudes; and organizational culture.	As research on digital mental health is still in its early stages, people with intellectual disabilities have often been excluded from developing, implementing, and evaluating digital interventions. Future development and research should actively involve people with intellectual disabilities and their professional carers in both development and evaluation. Assessing the accessibility and usability of digital mental health products for this group is essential.
Watfern	2019	A key finding was that participants working closely with a support worker were more engaged, as they could discuss content and receive help with activities. In contrast, those using the website independently often skipped content or left tasks incomplete. Participants saw the website as part of a wider support network involving family, support workers, and health professionals. An unexpected benefit was its ability to spark communication between people who might not otherwise interact. The website was also viewed as a safe space to explore mental health concerns without	People with intellectual disabilities face numerous barriers to mental health care, including limited services and challenges with communication and diagnosis. Digital mental health platforms could help reduce these barriers by offering accessible tools for communication and treatment. However, many are not designed with cognitive differences in mind. Accessible or inclusive	Important accessibility features include easy navigation, audio options (such as screen readers or audio files), videos, and interactive animations. Content should use Easy Read English, supported by meaningful

		judgement—especially important for those hesitant to identify with mental health issues or disability—and as a potential gateway to other support services.	design seeks to ensure that digital technologies are usable by people of all abilities.	images that clearly relate to the text. Some users preferred photographs of real faces, while others found cartoons or emojis easier to recognize. The login process should also be simple and straightforward.
Frielink et al.	2021	When service users, relatives as well as professionals are asked to illustrate examples of eHealth applications they knew, they mentioned a great diversity of eHealth applications, ranging from informational websites designed for people with intellectual disabilities to the use of social media, such as Facebook and YouTube, and the use of domotica/surveillance technology. In addition, participants indicated the use of eCommunication, and e-mail and video calls in particular, in the contact between service users and their family and support staff to be supportive.	In general, missing research on eHealth in people with intellectual disabilities mentioned as well as support delivered through eHealth can make service users less dependent on the available time and willingness of their support staff and relatives.	**Facilitating factors** for eHealth in supporting daily functioning include acknowledging that not everyone is willing to use eHealth, tailoring it to individual needs and abilities, involving all stakeholders (including relatives) from the start, ensuring users control their own data with strong security and clear privacy policies, sharing experiential knowledge, and meeting basic preconditions such as affordability, time, internet access, and suitable devices.<br><br>**Impeding factors** include privacy concerns, lack of or poor internet connection, high costs, insufficient IT support, and complex eHealth applications.
Vetter et al.	2022	Six main dimensions were inductively outlined, which are "interpersonal relationship", "organizations and communities", "healthcare system", "education", "digital interaction spaces", "politics", and "cultural contexts". The domain of digital spaces has a special role. This is found in each of the other domains. The use of digital interaction spaces offers a significant opportunity to teach and to promote health literacy. Too little knowledge about **digital health literacy** is assumed.	It is assumed that people with intellectual disabilities are disadvantaged by their cognitive limitations within a typical understanding of health literacy (focus on functional health literacy). There is little knowledge about the social context and people with intellectual disabilities, and research such as a review of evidence and how it is conducted for general health literacy is missing.	A more resource-oriented perspective should be considered. Beside others, specific (health) situations, social context and everyday life routines are important key factors for health literacy, which is considered as social practice.

Arachchi et al.	2023	Participants showed familiarity with health topics relevant to their daily lives. They were often prompted to seek information through conversations with doctors, family, or advertisements. An equal number searched for information about exercise and about doctors or hospitals. Some used both the internet and healthcare professionals interchangeably, while others relied on family, support workers, or community members for information. Examples included: “my mum is a nurse – my sister helps” and “when my sugar went high, my support workers looked on Google for ways to reduce it.” A few participants were not interested in searching for health information. They identified limiting factors that require attention from both designers and educators. Participants recognized the internet’s ability to meet their information needs quickly, suggesting that promoting safe internet use is more beneficial than overprotection.	Little is offered to designers of health information access that would inform them about the information interests, learning needs and accessibility requirements of people with intellectual disability. Moreover, strategies to satisfy the needs of people with intellectual disability in connection with technology use require further attention, also with close collaboration to see whether the requirements are met.	Introducing practical search examples could help participants understand how the Internet connects people with information. Those seeking support expressed interest in learning computer skills, asking for “step-by-step guidelines” and assistance.<br><br>Participants hoped future online health information would better meet their needs, requesting simpler language, clearer wording, reduced information overload, and blocked pop-up ads. Simplified explanations of complex terms, such as those provided by a HealthTranslator, could also be helpful.<br><br>Participants who can read and spell may benefit from search features like autocomplete and suggested keywords. Research shows that people with intellectual disabilities perform better with search engines featuring simple, uncluttered interfaces.
Dam et al.	2023	The resulting website prototype contains four main topics acquired with the focus groups: nutrition, medical specialization, patient information and first aid. The study shows the necessity of a compromise between content and usability as too much content or a too broad range of topics described in a website result in reduced usability since finding information becomes more difficult. While self-testing websites containing vast variety of health information targeting patients without intellectual disabilities, it was found that navigating websites with extensive content difficult as too specific information is convoluted. This is especially true if patients do not exactly	People with intellectual disabilities face major challenges understanding written information, which is often too complex. Communication between health professionals and patients can also be difficult, leading to misunderstandings and poorer health outcomes.	The website used easy-to-read language supported by images, covering topics from healthy recipes to health professions, chest compressions, and the Austrian electronic health record. An open-source WordPress theme was adapted

		know their search goals. A part of participants expressed frustration about repeating information, when other participants appreciate repeating information.	When creating a website, a key challenge is reducing text length while still providing essential information that supports understanding and improves health literacy.	according to key web accessibility guidelines. A balance between content and usability was necessary, as excessive content or too many topics can reduce usability by making information harder to find. Game-based learning, with clearly defined learning outcomes, could also be incorporated.
Savage	2023	Three participants take part. For two participants the combination of the health app with self-management intervention was effective in increasing the number of healthy food and drink items bought at the grocery store. Participants reported that they enjoyed using the app, scanning food and drink items was fun, and the goal-setting and self-monitoring components were easy to understand. They agreed that (a) they liked the Fooducate app, (b) they felt the app helped them keep track of their health, (c) the app helped them want to make healthier nutrition choices, (d) the app gave them information to help them be healthier, (e) the app could help others make healthier choices, and (f) they wanted to keep using the app after the project was over. But participants did not explore app features outside of the scanning feature.	While the use of mobile health technologies has increased, little is known about adults with disabilities' use of such health apps. Users with disabilities who use mobile health apps have described challenges and concerns about universal design and the need for additional support. For adults with intellectual disabilities, technology use with the addition of self-management strategies like goal setting, self-monitoring, and visual support interventions has been promising in promoting behavior change in other behaviors such as academic, exercise, and work-related tasks.	Adaptions are not described. During baseline, participants had access to the Fooducate app, visual supports to navigate the app. It was beneficial that this study was conducted in real-world contexts,
